# Toward precision neuroscience in Alzheimer’s disease: the role of multimodal AI

**DOI:** 10.3389/fnagi.2026.1903637

**Published:** 2026-07-30

**Authors:** Noelia Martínez-Molina, Sílvia Orte, Carolina Migliorelli, Vicent Ribas

**Affiliations:** Digital Health Unit, Eurecat Technology Center, Barcelona, Spain

**Keywords:** Alzheimer’s disease, biomarkers, computational neuroscience, digital health, multimodal AI

## Abstract

**Background:**

Alzheimer’s disease (AD) is increasingly understood as a biologically defined and heterogeneous continuum, requiring models that move beyond symptom-based diagnosis toward individualized risk prediction, stratification, and intervention.

**Objective:**

This mini-review outlines how precision neuroscience frameworks may support the characterization of AD by integrating multimodal biomarkers, systems biology, systems neurophysiology, digital health markers, and artificial intelligence (AI).

**Methods:**

We synthesize recent developments across multi-omics profiling, neuroimaging and electrophysiological biomarkers, AI-based speech analysis, and multimodal machine learning approaches, with emphasis on their potential contribution to biologically informed disease staging and personalized clinical decision-making.

**Results:**

Omics and systems biology approaches are expanding the characterization of molecular pathways involved in AD susceptibility, progression, and treatment response. Systems neurophysiology, including multimodal neuroimaging, electrophysiology, and whole-brain modeling, provides complementary markers of large-scale network disruption across the AD continuum. Digital health technologies, particularly AI-based speech analysis, offer scalable and ecologically valid tools for early risk enrichment and longitudinal monitoring. Multimodal AI models further enable the integration of heterogeneous clinical, molecular, imaging, genetic, and behavioral data into probabilistic representations of disease burden and progression. However, clinical translation remains constrained by interpretability, harmonization, validation, fairness, and accessibility challenges.

**Conclusion:**

Precision neuroscience offers a promising framework for reconceptualizing AD as a dynamically modeled and biologically stratified disorder. Future progress will depend on robust multimodal datasets, transparent AI methods, longitudinal validation, and equitable implementation strategies capable of supporting early detection, trial enrichment, and personalized prevention or treatment.

## Introduction

Alzheimer’s disease (AD) is increasingly conceptualized as a biologically defined continuum characterized by the progressive accumulation of extracellular amyloid-β (Aβ) plaques, tau neurofibrillary tangles and neurodegeneration that emerge years before the onset of overt clinical symptoms (Jack et al., 2018; Therriault et al., 2024). Accumulating clinical evidence supports the view of AD as a heterogeneous neurodegenerative disorder, reflected in differences in disease susceptibility, contributing risk factors, biomarker expression, longitudinal trajectories, clinical phenotypes, and responsiveness to intervention (Karran and Hardy, 2014; Jack et al., 2018; de Rojas et al., 2021). In addition, sex constitutes an important source of variability, shaping differences in disease risk, biomarker profiles, response to treatment and prognosis (Ferretti et al., 2018). In particular, women undergoing the menopausal transition appear to show an increased predisposition to AD relative to men of comparable age, which has been linked to alterations in amyloid-related and immune-inflammatory mechanisms, degeneration of cholinergic systems in the basal forebrain, and large-scale network dysfunction affecting brain communication and integration (Cavedo et al., 2018; Ferretti et al., 2018; Hohman et al., 2018; Vergallo et al., 2019). Importantly, these effects do not seem to be attributable solely to increased longevity or aging itself, but rather point toward a significant contribution of hormonal and endocrine changes associated with menopause (Christensen and Pike, 2015; Ferretti et al., 2018). Such heterogeneity poses major challenges for clinical characterization and patient stratification.

The characterization of biological markers has emerged as a central strategy for addressing the marked heterogeneity of AD. Over the last two decades, substantial advances in fluid and neuroimaging biomarkers have transformed the field, enabling the transition toward biologically grounded models of disease classification. In particular, fluid and plasma biomarkers provide scalable and minimally invasive alternatives to PET imaging, although they do not capture the spatial topology of the underlying brain alterations (Montoliu-Gaya et al., 2025). These efforts have culminated in the development of the Amyloid-β/Tau/Neurodegeneration [AT(N)] system, which stratifies individuals upon core pathophysiological changes in AD (Jack et al., 2016). Consistent with the principles of precision medicine (PM), the AT(N) framework aims to overcome the limitations associated with traditional symptom-based diagnostic approaches that rely primarily on clinical presentation (Jack et al., 2018). More recently, this model has been further expanded into the ATX(N) framework, in which the “X” component incorporates additional pathophysiological mechanisms beyond the canonical amyloid, tau, and neurodegeneration axes, including processes such as neuroinflammation and blood–brain barrier dysfunction.

This reconceptualization aligns with the precision neuroscience (PN) notion, a concrete case of PM, which according to the landmark announcement of the US PM Initiative can be defined as “prevention and treatment strategies that take individual variability into account” (Collins and Varmus, 2015). In its mature implementation within clinical settings, PN is expected to align with the principles of the “P4” medicine framework (Hood and Friend, 2011). This paradigm encompasses: (i) the identification and stratification of individuals according to their probability of developing disease-related pathology (predictive); (ii) the promotion of early detection and population-level screening strategies to enable timely intervention before substantial clinical deterioration occurs (preventive); (iii) the adaptation of therapeutic approaches to each individual’s biological, clinical, and psychosocial profile (personalized); and (iv) the development of patient-centered and data-driven care models that actively involve individuals through continuous monitoring, self-assessment, and individualized management strategies (participatory). In line with this, the integration of longitudinal digital phenotyping and personalized behavioral support strategies capable of capturing real-world disease dynamics and promoting modifiable risk reduction is of utmost importance.

**FIGURE 1:**
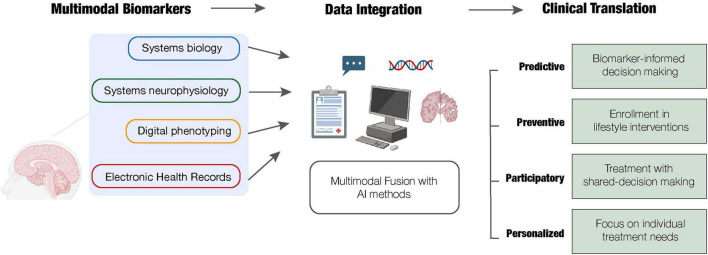
Conceptual framework for precision neuroscience in Alzheimer’s disease. Multimodal biomarkers derived from systems biology (e.g., multi-omics profiling), systems neurophysiology (e.g., neuroimaging and electrophysiology), digital phenotyping, and electronic health records are integrated through artificial intelligence (AI)-based multimodal fusion approaches to support clinical translation within precision neuroscience frameworks. This integrative architecture aligns with the principles of P4 medicine by enabling predictive biomarker-informed decision making, preventive enrollment in lifestyle and early-intervention strategies, participatory treatment through shared decision-making models, and personalized therapeutic approaches tailored to individual biological and clinical profiles. The framework illustrates how multimodal data integration may facilitate biologically grounded stratification and individualized management across the AD continuum. Icons were obtained from https://www.biorender.com/.

Following the subsequent AD PM Initiative (Hampel et al., 2019), in this mini-Review we propose a PN architecture based on the following converging pillars: systems biology, systems neurophysiology, digital health technologies, and data science applied to electronic health records (Figure 1). We present AD as a representative example of this framework given the substantial unmet clinical needs that continue to characterize the field, though it could be extended to other neurological and psychiatric disorders.

## Systems biology: multi-omics profiling

Omics disciplines – including genomics, epigenomics, transcriptomics, proteomics, metabolomics, and lipidomics – integrated within systems biology (SB) frameworks offer the potential to generate interpretable biological signatures spanning the full continuum of AD. SB represents a data-driven and integrative approach that examines biological processes as dynamic and interconnected molecular networks, enabling the simultaneous investigation of molecular interactions and their temporal organization at the systems level (Chen et al., 2012; Bassett and Sporns, 2017; Hampel et al., 2021). This direction in neurology is further motivated by the success of omics and SB approaches in oncology, where pathway-oriented and stage-specific therapeutic strategies have emerged in parallel with the development of PM paradigms.

Large-scale genomic studies, particularly genome-wide association studies (GWAS), have substantially advanced the understanding of the genetic architecture underlying AD, identifying numerous common variants associated with disease susceptibility. Beyond the well-established role of APOE ε4 as the strongest genetic risk factor for late-onset AD, recent meta-GWAS analyses have uncovered additional loci involved in amyloid processing, immune regulation, lipid metabolism, synaptic signaling, and neuroinflammation. These include risk-associated variants in genes such as SHARPIN, CHRNE, PRKD3/NDUFAF7, and IL34, as well as protective variants in APP, PLCG2, and APOE ε2, highlighting the complex and heterogeneous biological pathways contributing to AD pathogenesis (de Rojas et al., 2021). Polygenic risk scores (PRS), which aggregate the weighted effects of multiple susceptibility variants across the genome, have emerged as promising tools for translating genomic discoveries into clinically meaningful risk stratification. PRS approaches have demonstrated associations with conversion from mild cognitive impairment (MCI) to AD, neuropathological burden, and age at disease onset, while also enabling the identification of individuals at particularly high genetic risk, especially among APOE ε4 carriers. Importantly, recent findings (Chaudhury et al., 2019) indicate that PRS can discriminate clinically relevant differences in age at onset by several years, supporting their potential utility for early detection, patient stratification, and enrichment of prevention-focused clinical trials.

Epigenomics, which examines genome-wide regulatory modifications that influence gene expression without altering the underlying DNA sequence, provides a powerful framework for investigating interactions between genetic susceptibility and environmental exposures (Garrett-Bakelman et al., 2019). In this regard, recent work (Genon et al., 2026) has emphasized the importance of adopting an exposome-informed perspective to understand how environmental, lifestyle, biological, and sociodemographic factors interact dynamically across the lifespan to shape neurocognitive phenotypes, as well as vulnerability and resilience to AD.

Transcriptome-wide association studies can provide important insights into the spatial and temporal organization of molecular mechanisms underlying neurodegenerative disorders, helping to distinguish primary causal pathways from downstream biological consequences associated with emerging genetic and biological risk factors (Noori et al., 2021; Bhattacharya et al., 2026). Proteomics has likewise become a key approach for characterizing disease-related molecular pathways and for the discovery, validation, and clinical qualification of fluid biomarkers in AD (Johnson et al., 2020). Although still comparatively less developed than other omic domains, metabolomics and lipidomics offer the possibility of capturing highly individualized profiles of bioenergetic function, metabolic regulation, and lipid homeostasis, thereby contributing to the understanding of critical pathophysiological processes involved in AD (Varma et al., 2018).

## Systems neurophysiology: neuroimaging, electrophysiology and computational modelling

Systems neurophysiology aims to characterize how large-scale neural dynamics emerge across spatial and temporal scales by integrating structural and functional measures of brain activity, thereby enabling the generation of functional atlases spanning development, healthy aging, and disease (Hampel et al., 2023). One important example is the study of the brain hierarchical organization in cognitively normal young and aged individuals and its reconfigurations in AD.

In a recent multimodal study (Montaña-Valverde et al., 2025), we combined PET, structural MRI, diffusion MRI, and resting-state fMRI data from ADNI3 to characterize how the directed hierarchical organization of large-scale brain dynamics is reconfigured across the AD continuum. Using whole-brain generative effective connectivity and ecology-inspired trophic measures, we found that cognitively normal amyloid-β negative (Aβ−) individuals showed a stratified hierarchy in which somatomotor and frontoparietal regions acted as causal sources and the transmodal default mode network occupied an intermediate integrative position. Across disease progression, this architecture progressively flattened: higher-order cortical systems, including somatomotor, salience, control, and default mode networks, lost hierarchical influence. These hierarchical alterations were associated with amyloid burden, tau deposition, grey matter atrophy, and cognitive impairment, and further discriminated disease stages, including the preclinical distinction between cognitively normal individuals with and without amyloid positivity.

Multiscale whole-brain modeling approaches further extend systems neurophysiology by enabling the mechanistic integration of molecular pathology with large-scale brain dynamics (Patow et al., 2023). By incorporating regional Aβ and tau distributions derived from PET imaging into a biophysically informed whole-brain model, it was shown that Aβ exerted a predominant influence during early disease stages, particularly in MCI, whereas tau became the dominant driver in later AD stages. Importantly, the combined spatial distributions of Aβ and tau improved the modeling of empirical functional dynamics beyond homogeneous whole-brain models, supporting the notion that molecular pathology reshapes large-scale neural activity through distinct but interacting effects on excitatory and inhibitory neuronal populations.

Another prominent functional alteration in AD is the slowing of electro/magneto-encephalographic (E/MEG) activity, characterized by a redistribution of neural oscillatory power toward lower frequency bands (Babiloni et al., 2013). These electrophysiological abnormalities have been linked to cortical atrophy and to disrupted connectivity with key hub regions, including the hippocampus as well as occipital and posterior default mode network areas (Maestú et al., 2019). Recent MEG and computational whole-brain modeling studies further suggest that these alterations reflect a progressive disruption of brain criticality and excitation–inhibition balance, characterized by attenuated long-range temporal correlations, increased neuronal hyperexcitability, and a shift toward excitation-dominated supercritical dynamics across the AD continuum (Javed et al., 2025). Personalized neural mass models integrating PET and resting-state fMRI data reproduced hallmark AD spectral changes, including theta enhancement and alpha-band reductions, which became detectable already in cognitively normal Aβ+ individuals and after limbic tau involvement (Sanchez-Rodriguez et al., 2024). Importantly, these abnormalities were associated with medial temporal lobe atrophy, plasma p-tau and GFAP (Glial Fibrillary Acidic Protein) concentrations, cognitive decline, and longitudinal disease progression from MCI to AD, supporting their potential utility as early systems-level biomarkers of neurodegeneration. These changes are particularly relevant as potential neuroimaging markers, since EEG recordings are less costly and impose lower infrastructural demands than MRI, though they entail their own challenges in spatial resolution and cross-site harmonization.

## Digital health markers: AI-based speech analysis

Digitally enabled phenotyping approaches may provide access to a broad and ecologically valid spectrum of disease-related features that often remain insufficiently characterized during routine clinical visits (Kourtis et al., 2019). A key strength of digital health technologies lies in their portability and capacity for quantitative measurement, facilitating convenient, unobtrusive, and longitudinal collection of clinically relevant data. Digital health data may encompass a broad spectrum of physiological and behavioral metrics, ranging from autonomic biomarkers (e.g., heart rate, thermoregulation, cardiac rhythms, skin conductance, and oxygen saturation) to clinically relevant functional measures such as gait, motor activity, sleep, speech, and voice dynamics. Wearables and unobtrusive sensing technologies may complement conventional biomarkers by providing continuous insights into sleep, mobility, activity patterns, and daily functioning. Such technologies may additionally facilitate continuous patient engagement, self-management, and participatory preventive strategies across the AD continuum.

AI-based speech analysis has emerged as one of the most promising digital approaches for the early detection and biological stratification of AD. Spontaneous speech production constitutes a highly complex cognitive behavior requiring the coordinated integration of episodic memory, semantic processing, executive control, attention, and motor planning. Because these functions rely on distributed large-scale brain systems affected early in AD, subtle alterations in speech and language may precede clinically overt cognitive impairment by several years (He et al., 2025).

Digital speech paradigms can capture ecologically valid aspects of cognitive function while enabling large-scale and longitudinal monitoring. Story-recall and semantic fluency paradigms have proven especially informative, as they engage memory retrieval, semantic access, and discourse organization processes that are sensitive to early AD-related alterations. In particular, delayed story recall tasks provide naturalistic discourse samples from which semantic, lexical, and narrative features can be derived (Fristed et al., 2022). Prior studies have shown that alterations in proper-name recall and semantic retrieval are associated with amyloid burden even among cognitively normal individuals (Mueller et al., 2020), suggesting that speech abnormalities may emerge during preclinical stages of disease progression.

Recent work increasingly supports the biological relevance of speech-based digital phenotypes. König et al. (2026) evaluated the Speech Biomarker for Cognition (SB-C), an automated speech marker assessed across five European cohorts comprising 736 individuals with heterogeneous cognitive and biomarker profiles. The framework integrated speech features extracted from verbal learning and semantic fluency tasks administered remotely through app- and phone-based platforms. SB-C scores significantly differentiated cognitively normal and cognitively impaired participants, correlated with established neuropsychological measures including the Mini-Mental State Examination and Clinical Dementia Rating, and demonstrated moderate-to-high classification performance for cerebrospinal fluid Aβ and phosphorylated tau181 positivity. Importantly, biomarker differences were also observed among cognitively normal individuals with subjective cognitive decline, supporting the hypothesis that speech alterations may arise during the earliest biological stages of AD.

The rapid development of transformer-based language models (Vaswani et al., 2023) and self-supervised learning frameworks has further expanded the potential of speech markers in AD. Transformer architectures rely on self-attention mechanisms capable of modeling long-range contextual dependencies across speech and narrative sequences, enabling the extraction of latent multidimensional representations directly from raw language data. These approaches may capture disease-relevant alterations that remain undetectable using traditional handcrafted linguistic features or conventional cognitive screening tools (Rezaii et al., 2025). Multilingual transformer models such as XLM-RoBERTa additionally offer opportunities for adapting AI-based screening tools to linguistically diverse populations, an important consideration given that most current speech markers have been developed primarily in English-speaking cohorts, which creates a suboptimal benchmark for global populations, as structural linguistic differences dictate how dysfunctions manifest (Blasi et al., 2022).

Within PN frameworks, speech digital markers may function as a scalable first-line enrichment strategy preceding amyloidosis confirmation. Rather than replacing established molecular markers, speech markers may facilitate the identification of individuals at increased biological risk who could subsequently undergo more specific and resource-intensive testing. Such stepped diagnostic models combining behavioral digital phenotyping, blood-based biomarkers, and confirmatory specialist evaluation may reduce healthcare burden, improve referral prioritization, and facilitate earlier access to preventive interventions and disease-modifying therapies.

Despite these promising developments, several limitations continue to challenge widespread clinical implementation. Speech markers are highly sensitive to demographic, linguistic, educational, cultural, and socioeconomic variability, raising concerns regarding algorithmic fairness, generalizability, and bias. Moreover, although current studies demonstrate promising associations with Aβ (Fristed et al., 2022) markers, further validation in large biomarker-confirmed longitudinal cohorts will be necessary to establish disease specificity, prognostic utility, and reproducibility across neurodegenerative disorders.

## Data science and AI: multimodal models for data integration

Contemporary digital technologies are progressively enabling the large-scale acquisition of rich physiological and behavioral datasets, making the generation of “big data” an unavoidable feature of modern biomedical research and healthcare systems. The high dimensionality, heterogeneity, and scale of big data present substantial analytical challenges for traditional statistical approaches, necessitating more advanced computational and machine learning (ML) methodologies. AI-based computational frameworks are capable of deriving clinically meaningful representations from sparse, noisy, and multidimensional data acquired across diverse modalities and sources (Cirillo and Valencia, 2019).

Advanced ML and deep learning frameworks have become central tools for multimodal data integration, as they enable the characterization of complex nonlinear relationships spanning heterogeneous biological, clinical, imaging, and behavioral modalities while handling sparse and incomplete datasets more effectively than traditional statistical methods. Specifically, recent transformer-based models (Vaswani et al., 2023) have demonstrated the capacity to integrate demographic variables, medical history, neuropsychological assessments, neuroimaging, genetics, and fluid biomarkers into biologically meaningful latent representations of disease progression.

For example, Jasodanand et al. (2025) recently proposed a multimodal AI framework integrating clinical, neuroimaging, cognitive, genetic, and biomarker information across seven cohorts comprising more than 12,000 participants. Their transformer-based architecture predicted Aβ and tau PET positivity with robust performance while remaining resilient to incomplete feature availability. Importantly, the model jointly estimated Aβ and tau burden rather than treating these pathologies independently, reflecting their biological interdependence across disease progression. Moreover, the framework demonstrated alignment between AI-derived regional representations and known spatial patterns of tau deposition as well as postmortem pathology. These findings illustrate how multimodal AI may move beyond biomarker classification toward biologically informed probabilistic staging capable of supporting clinical trial enrichment, individualized risk estimation, and precision therapeutic stratification.

In addition, AI approaches may facilitate the identification of clinically meaningful subgroups within large and heterogeneous populations, revealing distinct genetic and biological profiles among individuals who otherwise appear to share similar clinical phenotypes or diagnostic categories (Jordan and Mitchell, 2015; Wang et al., 2021). Such approaches can be implemented through unsupervised learning techniques that autonomously detect hidden structures and relationships within high-dimensional datasets (Wang et al., 2021). The latent clusters or dimensions identified by these models may uncover biologically meaningful heterogeneity underlying superficially similar clinical phenotypes, with important implications for treatment response and disease progression (Jordan and Mitchell, 2015; Richards et al., 2019; Wang et al., 2021).

In clinical trials, unsupervised AI methodologies trained on clinical and biomarker datasets have already demonstrated the ability to predict therapeutic response in disorders such as depression (Tozzi et al., 2024) and AD (Hampel et al., 2020). The widespread implementation of electronic health records (EHRs) across healthcare systems has facilitated the large-scale collection and storage of real-world population-level clinical data in digital formats amenable to systematic computational analysis. AI applied to large-scale EHR databases has expanded capabilities for diagnosis, prognosis, and prediction of disease trajectories, providing clinically actionable insights that may support more informed and personalized decision-making (Wilkinson et al., 2020). Once adequately trained and validated, these models could provide a scalable and comparatively low-cost alternative to conventional population screening approaches by identifying individuals at elevated risk who may benefit from more specific confirmatory assessments.

## Potential limitations and challenges of AI for PN

Although AI approaches can achieve high predictive performance, their clinical implementation remains limited by several methodological and translational challenges. One major concern is the “black box” nature of many AI models, which restricts interpretability and hampers understanding of how specific variables influence predictions (Hampel et al., 2023). AI methods can capture the complexity of biological systems, but often fail to explain the mechanisms underlying this complexity, limiting clinical interpretability and decision-making (Rudin, 2019). However, there have been significant recent advances in post-hoc interpretability techniques that can approximate deep learning (DL) black-box models with simpler interpretable models that can be used to explain DL models (Rai, 2020). Additional challenges include the need for harmonized, well-curated, and multimodal datasets capable of integrating diverse biological and clinical dimensions across omics and neuroimaging domains (Dinsdale et al., 2022). These difficulties become particularly pronounced in large-scale multi-omics and systems biology frameworks. Furthermore, the successful translation of AI outputs into actionable interfaces depends heavily on defining the right clinical question at the outset (Hassan et al., 2023).

One major translational challenge is the meaningful integration of biological, neuropsychological, behavioral, and contextual data into clinically usable decision-support systems. In AD, multimodal approaches combining neuroimaging, molecular biomarkers, and genetics hold promise for identifying at-risk individuals, but their clinical adoption remains constrained by cost, technical complexity, and limited standardization. Moreover, AI systems must better incorporate socioeconomic and healthcare accessibility factors to support equitable and feasible clinical decision-making (Hampel et al., 2023). Ensuring accessibility, usability, and fairness of AI-driven PN tools will be essential to avoid widening existing healthcare inequalities.

## Conclusion

The convergence of multimodal biomarkers, systems neuroscience, and AI is progressively reshaping AD from a syndromic entity into a dynamically modeled and biologically stratified disorder. PN frameworks increasingly integrate molecular, neurophysiological, cognitive, and behavioral dimensions across the disease continuum. Given that the Lancet Commission (Livingston et al., 2024) has estimated that approximately 45% of dementia risk may be attributable to modifiable factors – including smoking, physical inactivity, and social isolation – the importance of identifying at-risk individuals for future cognitive decline cannot be overstated. Early identification may not only facilitate participation in emerging clinical trials, but also enable the implementation of targeted lifestyle interventions aimed at delaying symptom onset and reducing the societal, familial, and healthcare burden associated with AD.
